# Cardiac safety assessment of a novel recombinant bispecific antibody targeting the ether-à-go-go related gene 1 (hERG1)-β1 integrin macromolecular complex

**DOI:** 10.3389/fphar.2023.1237431

**Published:** 2023-09-12

**Authors:** Lorenzo Santini, Claudia Duranti, Chiara Palandri, Lucrezia Giammarino, Monica Musumeci, Lucia Carlucci, Chiara Capitani, Rossella Colasurdo, Fabio Recchia, Elisabetta Cerbai, Raffaele Coppini, Annarosa Arcangeli

**Affiliations:** ^1^ Department of Neurosciences, Psychology, Drug Research and Child Health, University of Florence, Florence, Italy; ^2^ Department of Experimental and Clinical Medicine, Section of Internal Medicine, University of Florence, Florence, Italy; ^3^ Institute of Life Sciences, Scuola Superiore Sant’Anna, Pisa, Italy; ^4^ Department of Medical Biotechnology, University of Siena, Siena, Italy

**Keywords:** cancer therapy, IKr, APD, QT prolongation, bispecific antibodies

## Abstract

**Introduction:** In the last decades, mounting evidence has pointed out the human ether-á-go-go–related gene (hERG1) potassium channel as a novel biomarker in human cancers. However, hERG1 sustains the cardiac repolarizing current IKr and its blockade can induce a prolonged QT interval at the ECG, which increases the risk of life-threatening arrhythmias. This represents a major hindrance for targeting hERG1 for antineoplastic therapeutic purposes. Based on our discovery that hERG1 resides in a macromolecular complex with the β1 subunit of integrin adhesion receptors only in tumors, and not in the heart, we generated (and patented WO2019/015936) a novel engineered, single chain, bispecific antibody in the format of a diabody (scDb-hERG1-β1). This antibody has been proven to target with high affinity the hERG1/β1 integrin complex and to exert a good antineoplastic activity in preclinical mouse models.

**Methods:** In the present study, we evaluated the cardiac safety of the scDb-hERG1-β1, determining the action potential duration (APD) of human cardiomyocytes, either atrial (from valve-disease patients) or ventricular (from aortic stenosis patients). Cardiac cells were incubated *in vitro* with i) the scDb-hERG1-β1, ii) the full length anti-hERG1 monoclonal antibody (mAb-hERG1) and iii) its single chain Fragment variable derivative (scFv-hERG1), from which the scDb-hERG1-β1 was assembled. All the tests were performed before and after treatment with the specific hERG1 blocker E4031. In addition, we have performed preliminary experiments, analyzing the effects of the scDb-hERG1/β1 *in vivo* measuring the QT interval length of the surface ECG after its injection intravenously in farm-pigs.

**Results:** The scDb-hERG1-β1 did not produce any lengthening of APD compared to control (vehicle) conditions, either in atrial or ventricular cardiomyocytes, whereas both the hERG1-mAb and the scFv-hERG1 produced a significant APD prolongation. The addition of E4031 further prolonged APD. The scDb-hERG1-β1 did not produce any alterations of the QT (and QTc) interval values, once injected intravenously in farm pigs.

**Discussion:** Overall, the above evidences plead for the cardiac safety of the scDb-hERG1-β1, suggesting that an application of this antibody for anti-cancer therapy will be untainted by cardiotoxicity.

## Introduction

In the last decades, mounting evidence has pointed out ion channels as novel biomarkers in human cancers (Lastraioli et al., 2015). Among them, the human *ether-á-go-go–related gene* (hERG1) potassium channel exerts a key role, being expressed in many types of human cancers (Arcangeli et al., 1995; Arcangeli, 2005), where its activity regulates different cancer hallmarks (Arcangeli et al., 2009; Arcangeli et al., 2023). In particular, in cancer cells hERG1 regulates slowly changing resting potential values (V_rest_), clamping them at rather depolarized values compared to normal cells (Schonherr et al., 1999), and triggers intracellular signaling pathways involved in cell survival, proliferation, motility and invasion, often exerting non-conductive roles (Becchetti et al., 2022). Overall, hERG1 could represent a novel cancer biomarker and a therapeutic target in different human cancers. However, hERG1 is physiologically expressed in the heart, where it sustains IKr and hence regulates cardiac repolarization (Vandenberg et al., 2012), which represents a major hindrance for its targeting for antineoplastic therapeutic purposes (Arcangeli and Becchetti, 2015). Searching for molecular and functional differences between the “cardiac” and the “tumor” hERG1, we found that, in tumors, hERG1 resides in a peculiar conformational state, strictly bound (less than 1 nm distance) to the β1 subunit of integrin adhesion receptors. This does not occur in the heart, where hERG1 is bound to classical accessory subunits, such as the potassium voltage-gated channel subfamily E regulatory subunit 1 KCNE1 (Becchetti et al., 2017). This finding strongly provides the possibility for the hERG1/β1 integrin complex to be considered as a druggable novel oncological target.

Based on that, we generated (and patented WO2019/015936) a novel engineered, single chain, bispecific antibody in the format of a diabody (*scDb-hERG1-*β*1*), which has been proven to target with high affinity the hERG1/β1 integrin complex in cancer cells (Duranti et al., 2021a; Duranti et al., 2021b). Blocking this complex switches the PI3K/Akt pathway off, which has a negative impact on cell growth, angiogenesis, and metastatic spread (Duranti et al., 2021b), when administered either alone or in combination with chemotherapeutic drugs, such as Gemcitabine for Pancreatic Cancer (Lottini et al., 2023). The diabody is characterized by desired pharmacodynamic parameters like rapid clearance (Duranti et al., 2021b), which contribute to making the *scDb-hERG1-*β*1* a potential candidate for targeted anticancer therapy.

In the present study, we assessed the cardiac safety of the *scDb-hERG1-*β*1* both *in vitro*, by measuring the action potential duration (APD) in human freshly isolated atrial and ventricular cardiomyocytes. In human cardiac cells, we compared the effects of *scDb-hERG1-*β*1* with those exerted by the full length anti-hERG1 monoclonal antibody (mAb-hERG1) and the anti-hERG1 single chain Fragment variable (scFv-hERG1) and the specific hERG1-blocker E4031. The safety of our novel antibody was preliminarily assessed *in vivo* in farm-pig, by measuring the QTc interval while injecting the *scDb-hERG1/*β*1* intravenously.

## Materials and methods

### Production of antibodies

#### mAb-hERG1 production

The mAb-hERG1 (MCK Therapeutics; Florence, Italy; patent number IT1367861) was produced by secretion from an hybridoma cell line (named A7) obtained from mice cell fusion and able to secrete the monoclonal antibody (Guasti et al., 2008; Lastraioli et al., 2020; Lottini et al., 2021). Hybridoma cells are routinely maintained in DMEM (Euroclone) supplemented with Glutamine 10 mM and 5% Fetal Clone serum (Euroclone). Supernatant is harvested and purified by affinity chromatography using AKTA Pure (Cytiva) mounted with Protein A columns (Cytiva).

#### scFv-hERG1 (single chain variable fragment) and scDb-hERG1-β1 (single chain diabody) production

The recombinant antibodies scFv-hERG1 and scDb-hERG1-β1 were produced as described in detail in (Duranti et al., 2018) and (Duranti et al., 2021a; Duranti et al., 2021b), respectively. Both the antibodies are recombinant antibodies encompassing the VH and VL portions of the mAb-hERG1, for the scFv-hERG1 and of two monoclonal antibodies directed against hERG1 (the mAb-hERG1) and β1 (the TS2/16 antibody), for the scDb-hERG1-β1. The two recombinant antibodies are both produced in *Pichia Pastoris* yeast transfected cells. Supernatant is harvested and purified exploiting the His Tag expressed by both proteins by affinity chromatography using AKTA Pure (Cytiva).

### Tissue processing

Septal specimens from patients with aortic stenosis and atrial samples from patients with mitral valve disease were collected from the operating room and rapidly washed in ice cold cardioplegic solution (see Table 1). Fresh tissue was kept in ice-cold cardioplegic solution and used to isolate single cardiomyocytes; in particular, the tissue was minced to small pieces (∼1 mm^3^) and subjected to enzymatic and mechanical dissociation to obtain viable single myocytes, as described before (Coppini et al., 2014). In brief, tissue chunks were minced to small pieces (∼1 mm^3^) and then transferred in a scraping device. The bathing solution was changed to Ca^2+^-free dissociation buffer (Isenberg, see Table 1) and heated to 37°C. Collagenase Type V and Protease Type XXIV (Sigma) were subsequently added, and tissue chunks digested for 2 h. During the digestion, the dissociation buffer was collected every 10 min from the scraping device and diluted with KB solution (see Table 1) at room temperature to stop the action of the enzymes. The myocytes were left to settle and then resuspended in 10 μM Ca^2+^ Tyrode solution (see Table 1). CaCl_2_ was added stepwise up to 0.6 mM. Cardiomyocytes were stored in this solution and used within 5 h.

**TABLE 1:** Composition of the solutions used in the study.

Solution	Content (in mmol/L)	pH adjustment
Cardioplegic	KH_2_PO_4_ 50, MgSO_4_ 8, HEPES 10, adenosine 5, glucose 140, mannitol 100, taurine 10	pH 7.4 with KOH
Ca-free solution	NaCl 120 KCl 10 KH_2_PO_4_ 1.2 MgCl_2_ 1.2 Glucose 10 Taurina 20 Pyruvate 5 Hepes 10	pH 7.2 with NaOH
KB	KCl 20, KH_2_PO_4_ 10, glucose 25, mannitol 5, L-glutamic acid monopotassium salt 70, *β*-hydroxybutyric acid 10, EGTA 10 and 2 mg/mL albumin	pH 7.2 with KOH
Tyrode	132 NaCl, 4 KCl, 1.2 MgCl_2_ 10 HEPES, and glucose 11 (+1.8 CaCl_2_ for experiments)	pH 7.35 with NaOH
Pipette solution	K-L-Aspartic acid 130, HEPES 10, Na_2_-ATP 5, Na_2_-GTP 0.1, EGTA 11, MgCl_2_ 2.0, CaCl_2_ 5.0	pH 7.2 with KOH

### Loading procedure

Cell suspension was left to settle for 5 min, then viable cardiomyocytes were resuspended in 1.8 mM Ca^2+^ Tyrode solution and incubated at 37°C for 1 h with the following antibodies, at the final concentrations reported in parentheses: hERG1-mAb (10 μg/mL), scFv-hERG1 (10 μg/mL) or scDb-hERG1-β1 (10 μg/mL). After exposure to one of the antibodies, viable cardiomyocytes were left to settle, resuspended in Tyrode solution, transferred to a small (0.5 mL) temperature-controlled recording chamber and finally used to perform patch clamp measurements.

### Patch clamp

Action potentials (APs) were measured in current-clamp mode using the ruptured-patch clamp configuration with a specific pipette solution (Table 1) (Coppini et al., 2013; Coppini et al., 2019). The experimental temperature was 36°C ± 2°C. APs were elicited with short depolarizing stimuli (<3 ms) at different frequency of stimulation (0.2, 0.5 and 1 Hz, 1 min at each frequency). Patch clamp recordings were performed before and after addition of E4031(5 µM). Main chamber solution is frequently cleaned by a fast-rate heated perfusion end placed at the chamber’s side, that allows fast washout of chemicals in the bath.

### Effects of scDb-hERG1- β1 on farm pigs

Determination of the dose to administer *in vivo* to farm pigs: we calculated the dose to inject into farm pigs based on the dose administered *in vivo* to mice (Duranti et al., 2021b). The chosen dose should respond to the NOEL (No observed adverse effect level) dose and was calculated according to (Nair and Jacob, 2016), taking into account a theoretical km of 3 for mouse and 35 for farm pig. Considering a farm pig with a weight of 40 kg, the total amount of antibody to use was of 27.4 mg. Two doses of the scDb-hERG1-β1 antibody were administered, at time 0 and after 1 h. Throughout the whole procedure, ECG was recorded, and blood pressure and peripheral pulse were also monitored. Animal sacrifice was performed with a potassium chloride solution to cause cardiac arrest. The procedures received the approval from the Italian Ministry of Health. To assess the impact of treatments on survival, animals were monitored.

## Results

### Effects of hERG1-mAb, scFv-hERG1 and scDb-hERG1-β1 on action potential duration (APD) in human cardiomyocytes from the left atrium

**FIGURE 1:**
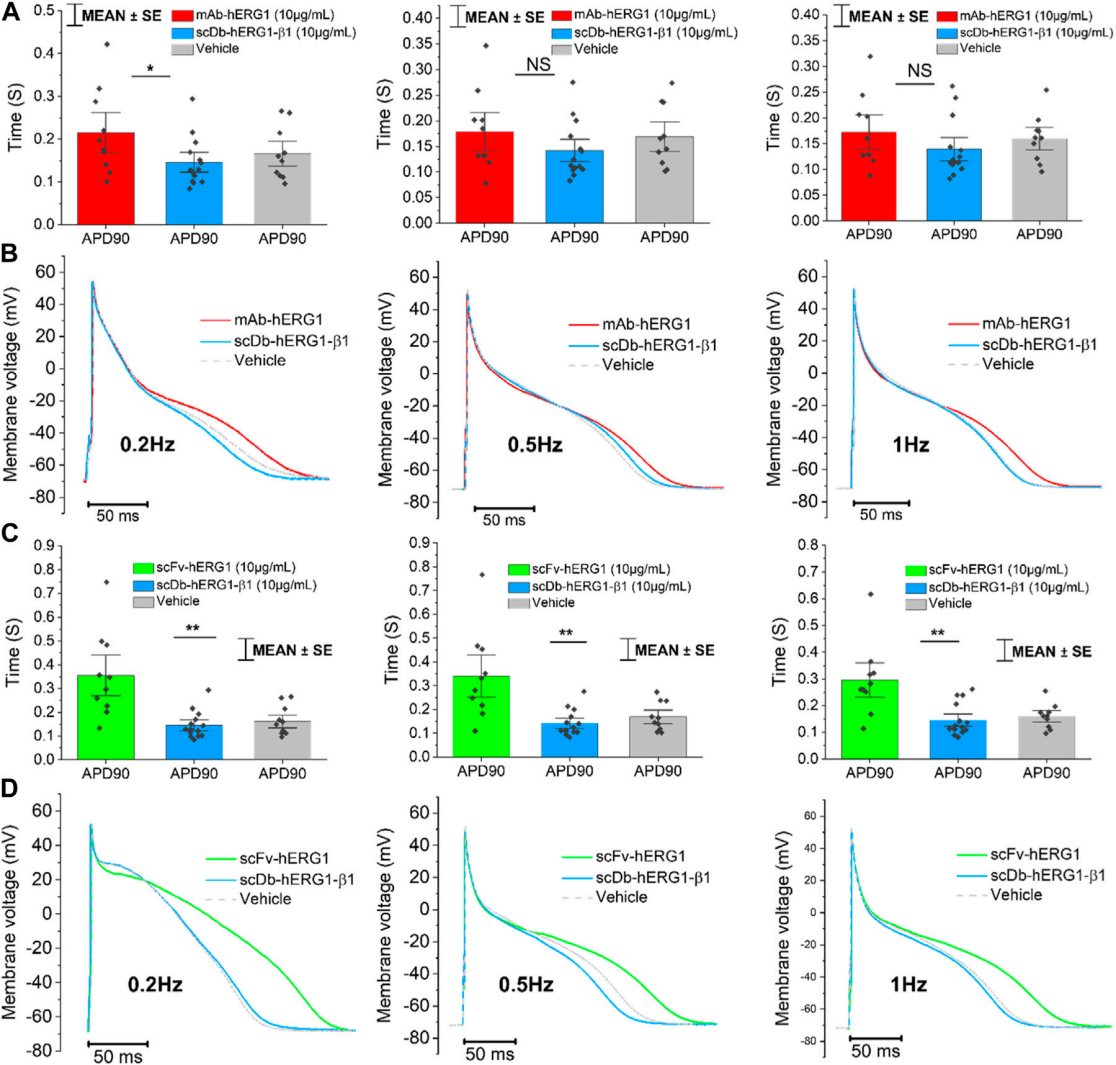
Action potential kinetics characterization of atrial cardiomyocytes isolated from valve-disease patients and incubated with hERG1-mAB, scFv-hERG1 and scDB-hERG1-β1. **(A)** Action potential duration at 90% repolarization (APD90%) recorded during stimulation at 0.2 Hz (left), 0.5 Hz (center) and 1 Hz (right) in human atrial cardiomyocytes incubated with hERG1-mAB (10 μg/mL), scDB-hERG1-β1 (10 μg/mL) or incubated with vehicle. **(B)** Superimposed representative action potential traces recorded in human atrial cardiomyocytes recorded during stimulation at 0.2 Hz (left), 0.5 Hz (center) and 1 Hz (right) and incubated with hERG1-mAB (10 μg/mL), scDB-hERG1-β1 (10 μg/mL) or vehicle. Mean ± SE from 24 cardiomyocytes (10 patients). * = *p* < 0.05; NS: not significant. **(C)** Action potential duration at 90% repolarization (APD90%) recorded during stimulation at 0.2 Hz (left), 0.5 Hz (center) and 1 Hz (right) in human atrial cardiomyocytes incubated with scFv-hERG1 (10 μg/mL), scDB-hERG1-β1 (10 μg/mL) or vehicle. **(D)** Superimposed representative action potential traces recorded in human atrial cardiomyocytes recorded during stimulation at 0.2 Hz (left), 0.5 Hz (center) and 1 Hz (right) and incubated with scFv-hERG1 (10 μg/mL), scDB-hERG1-β1 (10 μg/mL) or vehicle. Mean ± SE from 24 cardiomyocytes (10 patients). ** = *p* < 0.01.

Cardiomyocytes from the left atrium were isolated from valve-disease patients through an enzymatic and mechanical dissociation, then used for patch clamp experiments (ruptured patch clamping technique) to record AP, hence investigating the kinetics. After the isolation procedure, the cardiomyocytes’ suspension was incubated for 1 h in 1.8 mM Ca^2+^ Tyrode solution with hERG1-mAb (10 μg/mL), scFv-hERG1 (10 μg/mL) or scDb-hERG1-β1 (10 μg/mL) to test the effects of the antibodies on the electrical properties of atrial cardiomyocytes. hERG1-mAb significantly prolonged AP duration of left atrial cardiomyocytes compared to scDb-hERG1-β1, but this effect was evident only at 0.2 Hz pacing rate (Figure 1A left), while no changes were observed at faster stimulation rates (0.5 or 1 Hz, Figure 1A middle and right panels). Conversely, scFv-hERG1 significantly prolonged AP duration of atrial cardiomyocytes, at all pacing rates (Figure 1C left, middle and right). Interestingly, scDb-hERG1-β1 did not affect AP kinetics at any of the frequencies of stimulation we tested (Figures 1A–C).

### Effects of hERG1-mAb, scFv-hERG1 and scDb-hERG1-β1 on action potential duration (APD) in human cardiomyocytes from the left ventricle

**FIGURE 2:**
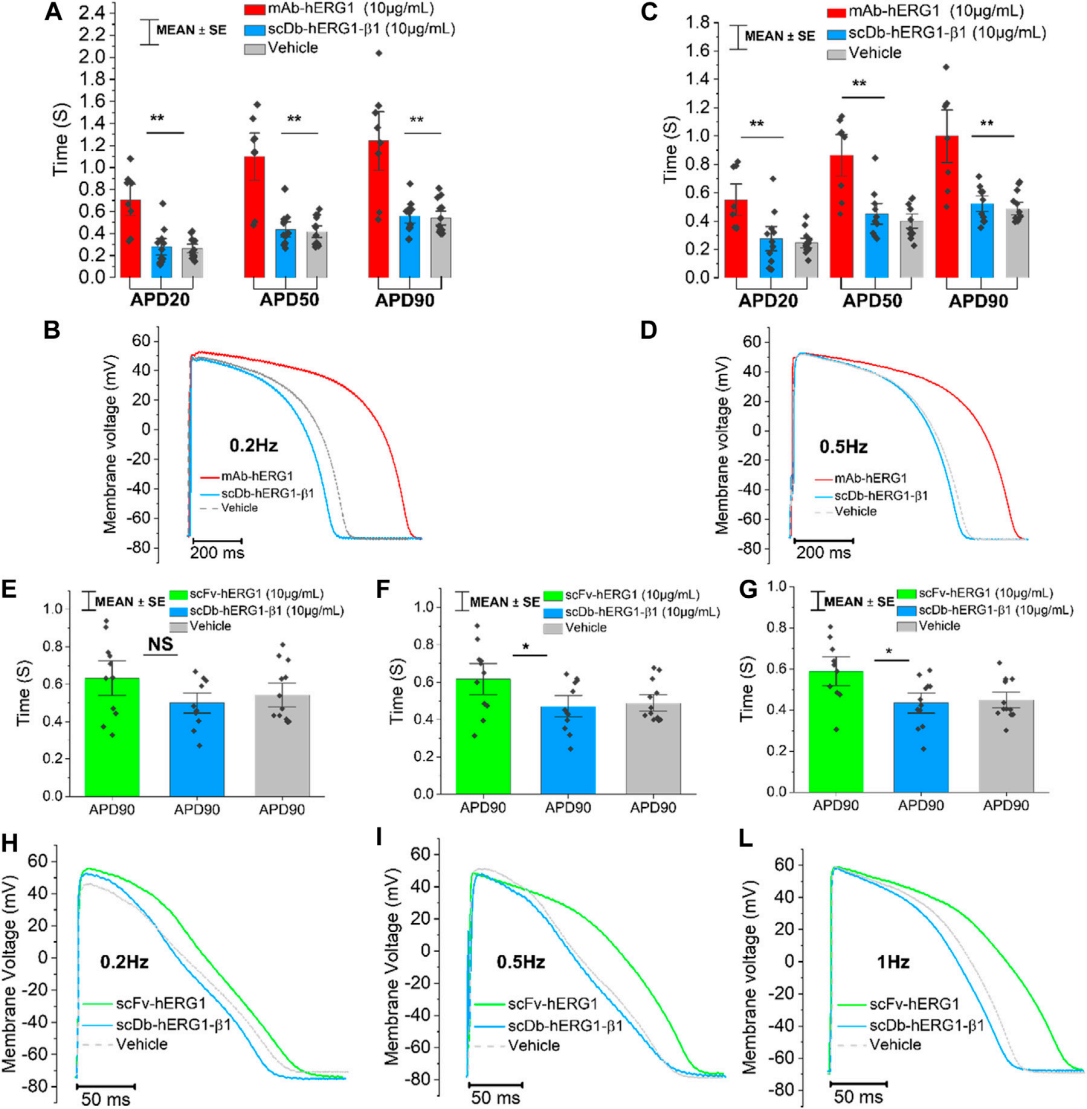
Action potential kinetics characterization of ventricular cardiomyocytes isolated from patients with aortic stenosis (AOS) and incubated with hERG1-mAB, scFv-hERG1 and scDB-hERG1-β1. **(A)** Action potential duration at 20%, 50% and 90% repolarization (APD20%, APD50% and APD90%) recorded during stimulation at 0.2 Hz in human ventricular cardiomyocytes incubated with hERG1-mAB (10 μg/mL), scDB-hERG1-β1 (10 μg/mL) or not incubated with Abs. **(B)** Superimposed representative action potential traces recorded in human ventricular cardiomyocytes during stimulation at 0.2 Hz and incubated with hERG1-mAB (10 μg/mL), scDB-hERG1-β1 (10 μg/mL) or vehicle. **(C)** Action potential duration at 20%, 50% and 90% repolarization (APD20%, APD50% and APD90%) recorded during stimulation at 0.5 Hz in human ventricular cardiomyocytes incubated with hERG1-mAB (10 μg/mL), scDB-hERG1-β1 (10 μg/mL) or vehicle. **(D)** Superimposed representative action potential traces recorded in human ventricular cardiomyocytes during stimulation at 0.5 Hz and incubated with hERG1-mAB (10 μg/mL), scDB-hERG1-β1 (10 μg/mL) or with vehicle. Mean ± SE from 24 cardiomyocytes (8 patients). * = *p* < 0.05; NS: not significant. **(E)** Action potential duration at 90% repolarization (APD90%) recorded during stimulation at 0.2 Hz in human ventricular cardiomyocytes incubated with scFv-hERG1 (10 μg/mL), scDB-hERG1-β1 (10 μg/mL) or with vehicle. **(F)** Action potential duration at 90% repolarization (APD90%) recorded during stimulation at 0.5 Hz in human ventricular cardiomyocytes incubated with scFv-hERG1 (10 μg/mL), scDB-hERG1-β1 (10 μg/mL) or not incubated with Abs. **(G)** Action potential duration at 90% repolarization (APD90%) recorded during stimulation at 1 Hz in human ventricular cardiomyocytes incubated with scFv-hERG1 (10 μg/mL), scDB-hERG1-β1 (10 μg/mL) or incubated with vehicle. **(H)** Superimposed representative action potential traces recorded in human ventricular cardiomyocytes during stimulation at 0.2 Hz and incubated with scFv-hERG1 (10 μg/mL), scDB-hERG1-β1 (10 μg/mL) or not incubated with Abs. **(I)** Superimposed representative action potential traces recorded in human ventricular cardiomyocytes during stimulation at 0.5 Hz and incubated with scFv-hERG1 (10 μg/mL), scDB-hERG1-β1 (10 μg/mL) or incubated with vehicle. **(L)** Superimposed representative action potential traces recorded in human ventricular cardiomyocytes during stimulation at 1 Hz and incubated with scFv-hERG1 (10 μg/mL), scDB-hERG1-β1 (10 μg/mL) or incubated with vehicle. Mean ± SE from 27 cardiomyocytes (7 patients). * = *p* < 0.05; ** = *p* < 0.01.

Cardiomyocytes from the left ventricle of patients with aortic stenosis were used to perform patch clamp experiments to evaluate AP kinetics. The cardiomyocyte suspension was incubated for 1 h in 1.8 mM Ca^2+^ Tyrode solution containing vehicle, hERG1-mAb (10 μg/mL), scFv-hERG1 (10 μg/mL) or scDb-hERG1-β1 (10 μg/mL). hERG1-mAb significantly prolonged APD of left ventricular cardiomyocytes with respect to vehicle (Figures 2A–C) at 0.2 Hz and 0.5 Hz. As previously observed in atrial cardiomyocytes (Figure 1C), scFv-hERG1 markedly prolonged the APD of ventricular cardiomyocytes compared to vehicle, at all pacing rates (Figures 2E–G). scDb-hERG1-β1 did not affect AP kinetics at any of the frequencies of stimulation tested (0.2, 0.5, 1 Hz); APD in cardiomyocytes incubated with scDb-hERG1-β1 was comparable to the vehicle and shorter than that measured in cells incubated with hERG1-mAb or scFv-hERG1 (Figures 2E–G).

### Effects of the specific hERG1 blocker E4031 on action potential duration (APD) in left ventricular cardiomyocytes incubated with scDB-hERG1-β1 and scFv-hERG1

**FIGURE 3:**
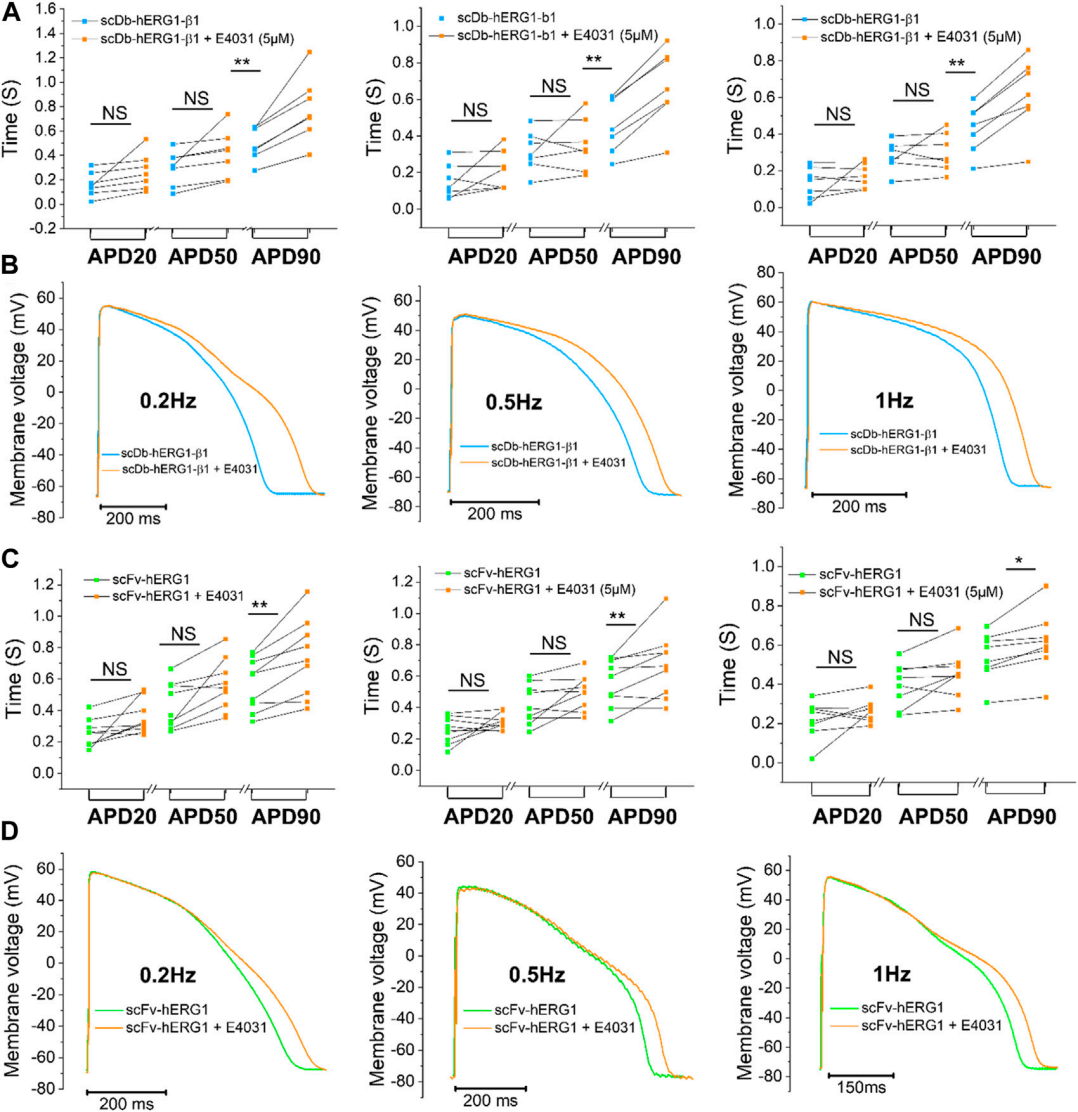
Effect of E4031 on action potential kinetics evaluated in ventricular cardiomyocytes isolated from patients with aortic stenosis (AOS) and incubated with scDB-hERG1-β1 and scFv-hERG1. **(A)** Action potential duration at 20%, 50% and 90% repolarization (APD20%, APD50%, APD90%) recorded during stimulation at 0.2 Hz (left), 0.5 Hz (center) and 1 Hz (right)in human ventricular cardiomyocytes incubated with scDB-hERG1-β1 (10 μg/mL), in absence and presence of E4031 (5 µM). **(B)** Superimposed representative action potential traces recorded in human ventricular cardiomyocytes during stimulation at 0.2 Hz (left), 0.5 Hz (center) and 1 Hz (right) and incubated with scDB-hERG1-β1 (10 μg/mL), in absence and presence of E4031 (5 µM). Mean ± SE from 10 cardiomyocytes (4 patients). ** = *p* < 0.01; NS: not significant. **(C)** Action potential duration at 20%, 50% and 90% repolarization (APD20%, APD50%, APD90%) recorded during stimulation at 0.2 Hz (left), 0.5 Hz (center) and 1 Hz (right) in human ventricular cardiomyocytes incubated with scFv-hERG1 (10 μg/mL), in absence and presence of E4031 (5 µM). **(D)** Superimposed representative action potential traces recorded in human ventricular cardiomyocytes during stimulation at 0.2 Hz (left), 0.5 Hz (center) and 1 Hz (right) and incubated with scFv-hERG1 (10 μg/mL), in absence and presence of E4031 (5 µM). Mean ± SE from 9 cardiomyocytes (4 patients). * = *p* < 0.01; ** = *p* < 0.01; NS: not significant.

**FIGURE 4:**
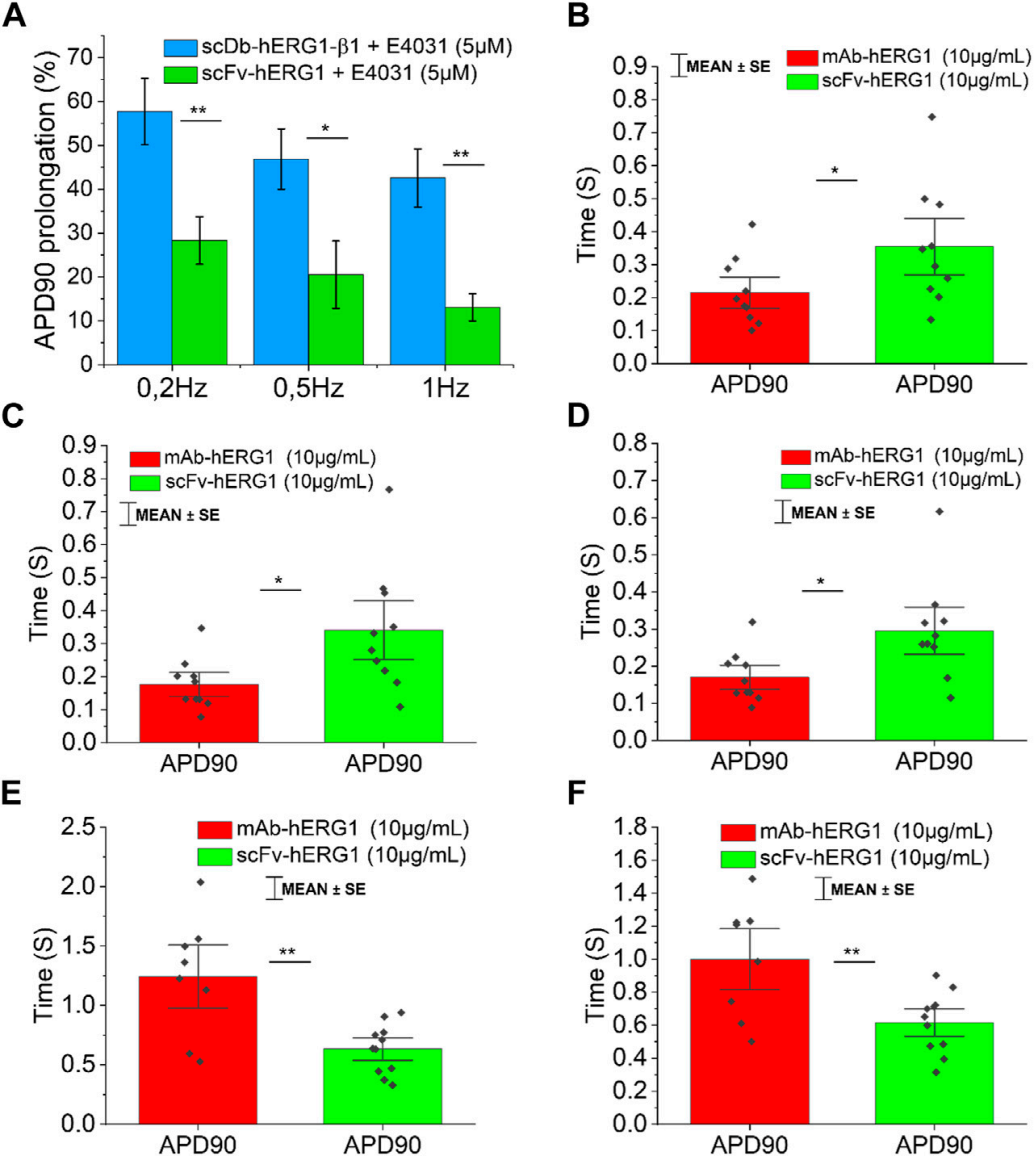
Comparison between the effect exerted by E4031 on action potential kinetics evaluated in ventricular cardiomyocytes isolated from patients with aortic stenosis (AOS) and incubated with scDB-hERG1-β1 or scFv-hERG1. **(A)** The figure shows the APD90 prolongation (%) promoted by the treatment with E4031 (5 µM) in ventricular cardiomyocytes incubated with scDB-hERG1-β1 (10 μg/mL) or scFv-hERG1 (10 μg/mL). The impact of E4031 on action potential kinetics is evaluated at different frequencies of stimulation: 0.2Hz, 0.5Hz, 1 Hz. * = *p* < 0.01; ** = *p* < 0.01; NS: not significant. **(B–D)** Action potential duration at 90 repolarization (APD90) recorded during stimulation at 0.2 Hz **(B)**, 0.5 Hz **(C)** or 1 Hz **(D)** in human atrial cardiomyocytes incubated with hERG1-mAB and scFv-hERG1 (10 μg/mL). * = *p* < 0.05. Mean ± SE from 20 cardiomyocytes (10 patients). **(E)** Action potential duration at 90 repolarization (APD90) recorded during stimulation at 0.2 Hz in human ventricular cardiomyocytes incubated with hERG1-mAB and scFv-hERG1 (10 μg/mL). **(F)** Action potential duration at 90% repolarization (APD90) recorded during stimulation at 0.5 Hz in human ventricular cardiomyocytes incubated with hERG1-mAB and scFv-hERG1 (10 μg/mL). ** = *p* < 0.01. Mean ± SE from 19 cardiomyocytes (10 patients).

To confirm whether the prolongation of AP duration was the direct consequence of hERG1 block, the specific hERG1 blocker E4031 (5 µM) was used as positive control for APD lengthening. As expected, E4031 treatment induced AP prolongation in left ventricular cardiomyocytes incubated with scDB-hERG1-β1 (Figure 3A left-center-right) as well as in ventricular cells treated with scFv-hERG1 (Figure 3C left-center-right). The prolongation exerted by E4031 is evident at all frequencies of stimulation investigated (0.2, 0.5, 1 Hz). The AP prolongation exerted by E4031 was larger in cardiomyocytes incubated with scDB-hERG1-β1 compared to scFv-hERG1 (Figure 4A). This is in line with the idea that scDB-hERG1-β1 does not bind hERG1 channels while scFv-hERG1 blocks part of them, thus reducing the effects of pharmacological channel block by E4031. Finally, we compared the electrophysiological effect of hERG1-mAb and scFv-hERG1 in left atrial cardiomyocytes from aortic stenosis patients. Both antibodies slow AP kinetics compared to scDB-hERG1-β1 but the most consistent prolongation of AP duration is exerted by scFv-hERG1 (Figures 4B–D), at all pacing rates evaluated (0.2, 0.5, 1 Hz). The comparison of the electrophysiological effect of hERG1-mAb and scFv-hERG1 was investigated also in left ventricular cardiomyocytes from aortic stenosis patients. In this case, both antibodies slow AP kinetics compared to scDB-hERG1-β1 but the most consistent prolongation of AP duration is exerted by hERG1-mAb (Figures 4B–D), at all pacing rates evaluated (0.2, 0.5 Hz).

### Effects of scDb-hERG1- β1 administered i. v. in farm pigs

**FIGURE 5:**
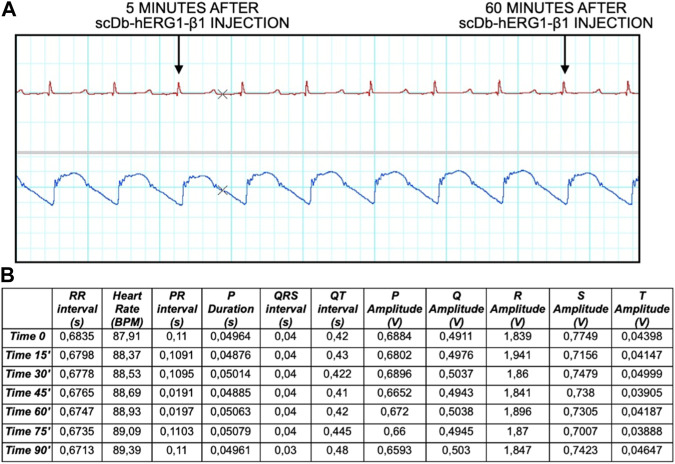
ECG trace and data analysis at time 0, 15, 30, 45, 60, 75, 90 min. **(A)** The ECG trace shows different time points 5 and 60 min after injection of the antibody. **(B)** Data analysis showing different parameters: RR interval (s), Heart Rate (BPM), PR interval (s), P Duration (s), QRS interval (s), QT interval (s), P Amplitude (V), Q Amplitude (V), R Amplitude (V), S Amplitude (V), T Amplitude (V).

Finally, we have performed preliminary experiments, in which the scDb-hERG1-β1 antibody was administered, as described in the Materials and Methods section, via catheter, at the dose of 0.69 mg/kg. This dose corresponds to the therapeutic dose of the antibody previously used in mouse models with either colorectal or pancreatic cancer (Duranti et al., 2021b), after the appropriate species conversion (Nair and Jacob, 2016). In particular, we evaluated ECG traces, mainly focusing on QT intervals, at different time intervals (after 5 min up to 1 h post injection). No alterations of the ECG trace were observed at all the times tested, in particular we found no alterations of the QT (and QTc) interval values. No signs of toxicity were observed neither in the ECG nor in the blood pressure and pulse, as reported in Figure 5A both after 5 min and 60 min (final time after injection). Data analysis is reported in Figure 5B showing no signs of alteration in the ECG trace, especially in the duration of QT interval, where hERG1-mediated current (IKr) plays a major role.

## Discussion

The present paper was aimed at testing the cardiac safety of a novel recombinant bispecific antibody targeting the hERG1/β1 integrin complex, developed in the form of a diabody: scDb-hERG1-β1. Both human atrial and ventricular cardiomyocytes were used for patch-clamp measurements. In addition to the scDb-hERG1-β1, the effects of two anti-hERG1 antibodies, the hERG1-mAb and the scFv-hERG1 (i.e., the two antibodies from which the scDb-hERG1-β1 was derived), were tested on the electrical properties of the cardiac cell. In particular, all the antibodies were tested for the AP features, before and after E4031 treatment. No changes in APD were observed in cells incubated with the scDb-hERG1-β1, compared to control (vehicle) conditions. On the contrary, both the hERG1-mAb and the scFv-hERG1 produced a prolongation of APD. This was evident at all pacing rates investigated (0.2–0.5, 1 Hz). To confirm that the prolongation of AP duration was the direct consequence of hERG1 block, the specific hERG1 blocker E4031 was used as positive control for APD lengthening. As expected, E4031 treatment induced AP prolongation, confirming the presence of a preserved hERG1 current in isolated cells. The safety of the scDb-hERG1-β1 was confirmed *in vivo* in farm-pig, by measuring the QTc interval after the i.v. administration of the diabody.

The human ether-à-go-go-related gene 1 (hERG1) was discovered, in mid-nineties, to represent the pore forming subunit of the rapid delayed rectifier K+ current IKr (Curran et al., 1995). Hence hERG1 significantly contributes to action potential (AP) repolarization and to set the duration of the QT interval of the surface electrocardiogram (ECG). Impairment of hERG1 function through either gene mutation or pharmacological blockade by diverse drugs in clinical use causes a prolongation of APD, which is mirrored by a prolonged QT interval at the ECG thus increasing the risk of life-threatening arrhythmias (Vandenberg et al., 2012). Many common medications that block hERG1 channels as an unintended side effect can also increase arrhythmic risk. Indeed, several drugs were withdrawn from the market, or their approval for clinical use was severely restricted in the past decades, Therefore, routine preclinical screening for hERG1 block has become mandatory for any newly developed compound, in the early phases of candidate screening (Vandenberg et al., 2012; Yue et al., 2015).

A parallel line of evidence, first produced in the same 1995 (Arcangeli et al., 1995), pointed out that hERG1 channels are also aberrantly expressed in several types of human cancers where they exert diverse, relevant regulatory activities on cancer cell behavior (Arcangeli, 2005; Arcangeli et al., 2009). Hence, hERG1 represents a novel cancer biomarker (Becchetti et al., 2022; Arcangeli et al., 2023). However, targeting hERG1 is hard to pursue due to the above pharmacological hindrances. A possibility to overcome such constrains came from the discovery that in cancer hERG1 forms a macromolecular complex with the β1 subunit of integrin receptors (Becchetti et al., 2017). The complex is not present in the heart, where hERG1 is bound to classical accessory subunits (Becchetti et al., 2017). Based on this evidence, our group developed several anti-hERG1 antibodies, and in particular a bispecific antibody, in the format of a diabody, the scDb-hERG1-β1, which specifically targets such complex (Duranti et al., 2021a; Duranti et al., 2021b).

Interestingly, our anti-hERG1 antibodies bind the channel in the same protein region that is recognized by pathological autoantibodies isolated from Anti-RO/SSA-positive patients with systemic sclerosis and other autoimmune rheumatologic diseases (Guasti et al., 2008; Lazzerini et al., 2016). The presence of such antibodies in the serum of patients is associated with long-QT syndrome and a greatly increased risk of torsade-de-pointes and other malignant ventricular arrhythmias (Chadwick and Goode, 2005; Gasparoli et al., 2015). Overall, these data clearly suggest that the scDb-hERG1-β1 is incapable of binding hERG1 channels in cardiac cells, further stressing the specificity of the diabody for the hERG1-β1 integrin complex, which is only found in cancer cells (Becchetti et al., 2017). In isolated guinea pig myocytes, hERG1 blockers slowed IKr deactivation, but only shortened APD in these myocytes at a high (30 μM) concentration or at lower concentrations after pretreatment with the IKr blocker dofetilide (Gasparoli et al., 2015). *Ex vivo* evaluation in Langendorff-perfused, isolated guinea pig hearts, revealed that the amplitude of T-waves was increased, prolonged the PR interval, and shortened the QT interval. Notably, farm-pig cardiomyocytes express hERG1 in large amounts, and specific hERG blockers (such as E4031) have been shown to significantly prolong QTc *in vivo* (Hegyi et al., 2020).

Bispecific antibodies (bsAbs) have significant advantages over intact antibodies especially for possible therapeutic and imaging applications. A basic bsAb is formed by one heavy-light chain pair from one antibody and another heavy-light chain pair from another antibody. Chimeric quadromas have species-restricted heavy-light chain pairing. Other examples of bsAbs are the constructs with their fragments connected by peptide chains, such as bispecific T cell engagers (BiTE) molecules, thereby avoiding random association of the chains. Nowadays, in oncology, there are more than 50 bsAbs in clinical trials exploiting different mechanisms of action, among which the engagement of immune cells with tumor cells, delivering of payload and blocking of signaling pathways. Two bispecific antibodies have already been approved, Catumaxomab and Blinatumomab, targeting EpCAM and CD3 and CD19 and CD3 in malignant ascites and acute lymphoblastic leukemia, respectively (Suurs et al., 2019). The scDb-hERG1-β1 has proven effective in targeting the hERG1/β1 integrin complex in cancer cells with high affinity (Duranti et al., 2021b). Blocking this complex switches the PI3K/Akt pathway off and this has a negative impact on cell growth, angiogenesis and metastatic progression. It is worth noting that the scDb-hERG1-β1 antibody did not show any staining, either IF or IHC, in human cardiac myocytes, even when its concentration was increased from 20 to 200 mg/mL. Given such published evidence, we have decided not to repeat the experiment. Moreover, we have taken into account that recovering the cells after such strong experimental procedures could bias further assays. The diabody is also characterized by desirable pharmacodynamic parameters, such as rapid clearance (Becchetti et al., 2017), which contribute to making the scDb-hERG1-β1 a potential candidate for targeted therapy. The antibody we have presented is a bispecific antibody which has been characterized showing a very favorable half-life of roughly 13.5 h which is not so common for naked bispecific antibodies, usually having a short half-life, the latter representing a limitation for therapeutic uses (Duranti et al., 2021b). Moreover, it is worth noting that there are no examples of bispecific antibodies directed against ion channels and used for therapeutic purposes, configurating the scDb-hERG1-β1 as a novel molecule in this field (Duranti and Arcangeli, 2019). After a proper pre-clinical characterization which will encompass, traditionally, repeat dose toxicology studies in rodents and non-rodents follow a standard design with assessment for clinical signs, bodyweight, food consumption, toxicokinetic, ophthalmoscopy, clinical pathology (hematology, clinical chemistry and urinalysis), organ weights, macroscopic examination and histopathology, along with electrocardiogram (ECG), such antibodies put themselves in the pipeline for clinical trials (Baldrick, 2011).

Overall, the evidence provided in the present paper pleads for the cardiac safety of the scDb-hERG1-β1, suggesting even if with preliminary data, that an application of this antibody to anti-cancer therapy will be untainted by cardiotoxicity, preventing a pathological prolongation of repolarization that could lead to the long QT type 2 (LQT2) syndrome and the related fatal arrhythmia.

## Data Availability

The raw data supporting the conclusion of this article will be made available by the authors, without undue reservation.
