# Supplementary material for: Cardiac safety assessment of a novel recombinant bispecific antibody targeting the ether-à-go-go related gene 1 (hERG1)-β1 integrin macromolecular complex
Source: Front Pharmacol. 2023 Sep 12;14:1237431. doi: 10.3389/fphar.2023.1237431 (PMC10520717; doi:10.3389/fphar.2023.1237431)
Supplement: Supplementary file 1 [file Table1.DOCX]

**Supplementary Material**


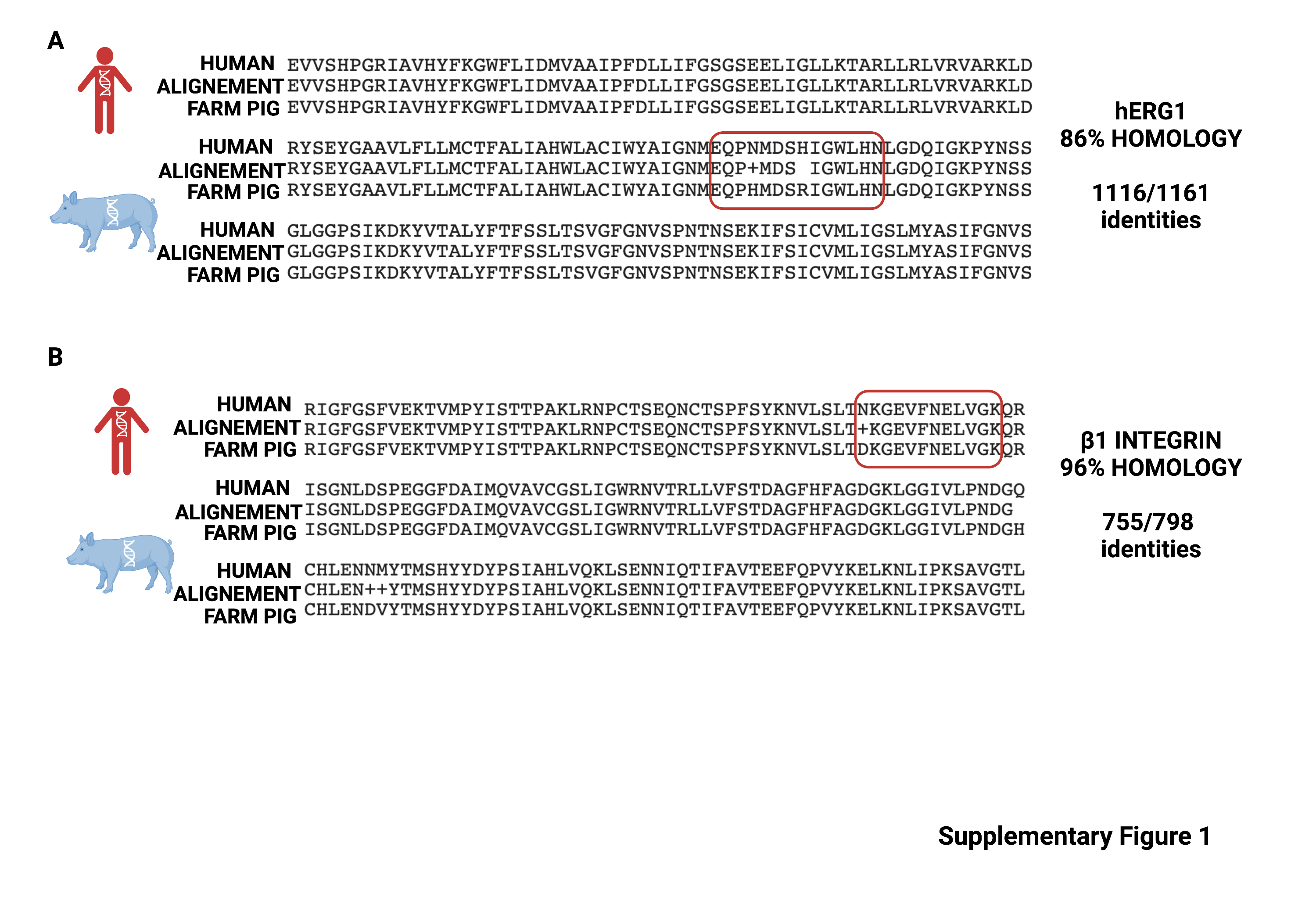


**Supplementary Figure 1. Aminoacidic sequence alignment showing the homology between the human and farm pig hERG1 and β1 integrin proteins, respectively. (A)** The first line shows human aa sequence, the second line shows the overlapping between the first and third line, the latter shows the mini pig aa sequence. In the red square is highlighted the epitope recognized by the one arm of the scDb-hERG1-β1, directed against hERG1. **(B)**
